# The Role of Autophagy in Tumor Immunology—Complex Mechanisms That May Be Explored Therapeutically

**DOI:** 10.3389/fonc.2020.603661

**Published:** 2020-12-01

**Authors:** Alana Serrano Campelo de Souza, Letícia Boslooper Gonçalves, Ana Paula Lepique, Patrícia Savio de Araujo-Souza

**Affiliations:** ^1^ Laboratório de Imunogenética e Histocompatibilidade (LIGH), Departamento de Genética, Setor de Ciências Biológicas, Universidade Federal do Paraná (UFPR), Curitiba, Brazil; ^2^ Programa de Pós-graduação em Genética, Departamento de Genética, Universidade Federal do Paraná (UFPR), Curitiba, Brazil; ^3^ Laboratório de Imunomodulação, Departamento de Imunologia, Instituto de Ciências Biomédicas, Universidade de São Paulo, São Paulo, Brazil

**Keywords:** macroautophagy, tumor microenvironment, antitumor immunity, tumor immune evasion, onco immunology

## Abstract

The tumor microenvironment (TME) is complex, and its composition and dynamics determine tumor fate. From tumor cells themselves, with their capacity for unlimited replication, migration, and invasion, to fibroblasts, endothelial cells, and immune cells, which can have pro and/or anti-tumor potential, interaction among these elements determines tumor progression. The understanding of molecular pathways involved in immune escape has permitted the development of cancer immunotherapies. Targeting molecules or biological processes that inhibit antitumor immune responses has allowed a significant improvement in cancer patient’s prognosis. Autophagy is a cellular process required to eliminate dysfunctional proteins and organelles, maintaining cellular homeostasis. Usually a process associated with protection against cancer, autophagy associated to cancer cells has been reported in response to hypoxia, nutrient deficiency, and oxidative stress, conditions frequently observed in the TME. Recent studies have shown a paradoxical association between autophagy and tumor immune responses. Tumor cell autophagy increases the expression of inhibitory molecules, such as PD-1 and CTLA-4, which block antitumor cytotoxic responses. Moreover, it can also directly affect antitumor immune responses by, for example, degrading NK cell-derived granzyme B and protecting tumor cells. Interestingly, the activation of autophagy on dendritic cells has the opposite effects, enhancing antigen presentation, triggering CD8^+^ T cells cytotoxic activity, and reducing tumor growth. Therefore, this review will focus on the most recent aspects of autophagy and tumor immune environment. We describe the dual role of autophagy in modulating tumor immune responses and discuss some aspects that must be considered to improve cancer treatment.

## Introduction

According to the Cancer Immune Edition hypothesis, tumor and immune cell interactions go through three phases: elimination, equilibrium, and evasion. During cancer development, the immune system recognizes molecular changes in transformed cells and eliminates most or all of them, avoiding tumor progression. Genetic alterations that cause cell transformation generate neoantigens for immune recognition, leading to T lymphocyte activation, which can prevent tumor outgrowth, through cytotoxic activity and interferon-gamma (IFN-*γ*) signaling (1, 2). At the same time, less immunogenic mutations or mutations that lead to loss of the antigen recognized by the immune system allow tumor cells to escape from elimination mechanisms. As genetic alterations accumulate, generating oncogenes and preventing the expression of tumor-suppressor genes, transformed cells gain proliferative advantages, and again escape immunosurveillance, leading to tumor progression (3, 4).

The interplay between tumor and other cells composing the tumor microenvironment (TME) is determinant for tumor growth, maintenance, metastasis, and response to therapy. TME is composed of stromal cells (fibroblasts, pericytes, mesenchymal and endothelial cells), extracellular matrix (ECM), and immune cells, such as natural killer (NK) cells, tumor-associated macrophages (TAMs), myeloid-derived suppressor cells (MDSCs), and T and B lymphocytes. During cancer progression, tumor cells display genetic and phenotypic diversity, changing cellular metabolism and, consequently, the TME (5, 6).

Generally, TME displays low levels of oxygen and nutrients, and high production of reactive oxygen species (ROS), crucial factors for autophagy activation. Autophagy is a natural cellular survival process, usually activated to maintain cellular homeostasis (7, 8). Despite that, recent studies have suggested that autophagy is also important for cancer development and progression, neurodegenerative and infectious diseases, once it can affect immune cells and modulate immune responses (9–11).

In this review, we will present the major mechanisms by which the immune system interferes in the TME, and how autophagy can influence it. Then we will focus on the modifications of cancer immune responses in TME influenced by autophagy and how it can affect cancer therapy.

## Cancer Immune Response and Tumor Microenvironment

Besides the TME elements already mentioned, soluble molecules as cytokines, metabolites, and inflammation mediators also contribute to the interaction among the cellular elements. These biochemical signals orchestrate cell death, proliferation, survival, and other cells recruitment. Leukocyte activity is essential in cancer progression (reviewed in 12). Since Rudolph Virchow has described the presence of lymphoreticular infiltrate in human tumors, it was discovered that leukocytes play an essential role in tumor progression, either by eliminating tumor cells or by facilitating progression and growth (13).

Immune responses can inhibit tumor growth and even eliminate tumor cells completely. However, chronic inflammation is considered a risk factor for many types of cancers (reviewed in 12). An interesting example of this dual role is HIV infection, AIDS (Acquired Immune Deficiency Syndrome), and cancer risk. In the early ‘90s, patients with AIDS were at high risk of Kaposi sarcoma and non-Hodgkin lymphoma development, in part due to cellular and molecular mechanisms, but in large part due to immunodeficiency. Highly active antiretroviral therapy (HAART) decreased AIDS-related cancer (14) by increasing T lymphocyte levels and consequently immune responses. Still, HAART treated HIV infected patients display chronic inflammation and early aging, with increased plasma levels of interleukin-(IL)6 and C reactive protein, which have a role in carcinogenesis. Indeed, HAART treated patients display increased risk to develop AIDS-unrelated cancer, such as cervical, lung, anal cancer, and Hodgkin lymphoma (14, 15).

The immune responses mediated by NK cells, CD8^+^ T lymphocytes, and CD4^+^ T helper (Th) 1 and Th17 lymphocytes are considered cytotoxic responses. These cells can control tumor growth through either directly killing tumor cells, as NK and CD8^+^ T cells, or indirectly, in the case of CD4^+^ T cells, which secrete cytokines capable of activating other effector leukocytes (16–18).

NK cells and CD8^+^ T lymphocytes are *bona fide* cytotoxic cells. NK cells are innate lymphoid cells that recognize target cells through activating and inhibitory receptors. The signaling triggered by these sets of receptors determines the cytotoxic activity. Among the inhibitory receptors, there are the killer immunoglobulin-like inhibitory receptors (KIRs), which recognize human leukocyte antigen (HLA) class I molecules and CD94/NKG2A, which specifically binds to the non-classical HLA-E molecule. The last one causes NK inhibition to ensure that normal cells cannot be lysed. However, transformed cells that downregulate the HLA-I surface molecules are not able to inhibit NK cells. The stimulatory receptors bind to stress-inducible molecules in the target cell surface, as sialic acid, Fc*γ*, adhesion molecules, and others, to trigger cytotoxic activity. Displaying a different strategy for target recognition, CD8^+^ T lymphocytes activation depends on TCR (T cell receptor) binding to specific antigens presented by classical HLA-I molecules in target cells (19). In spite of the different development and recognition receptors, both NK and CD8^+^ T cells display similar cytotoxic mechanisms, leading to the activation of cell death pathways in cancer cells (20).

Th1 and Th17 cells can either assist in CD8^+^ T lymphocytes and dendritic cells (DCs) activation, through CD40L signaling, and cytokine secretion as IL-2, or activate other effector cells, such as macrophages, neutrophils, NK cells through IFN-*γ* and tumor necrosis factor-alpha (TNF-α). Moreover, TNF-α, through its receptor, can trigger cell death, and IFN-*γ* and cytokines secreted by Th17 cells, through activation of stromal cells, can stimulate ROS production and neutrophils, enhancing the cytotoxic effects on cancer cells (21).

These antitumor responses are counteracted by tolerogenic responses, enabling tumor growth. There are several known immune escape mechanisms. Chemokines secreted by cells in the TME favors the recruitment of MDSCs and regulatory T cells (Treg), well-characterized suppressors of effector T lymphocytes function. Moreover, it is well known that cancer cells display reduction in antigen presentation potential, decreasing tumor cell recognition by CD8 T lymphocytes. One classic example, from a virus associated cancer is the HPV E7 oncoprotein, which binds to interferon regulatory factor 1 (IRF1) in the IFN type I (IFN-I) signaling pathway, and recruits histone deacetylase (HDAC) to the promoter sequences responsive to IRF1, repressing genes that otherwise would be transcribed in response to the virus (22). IFN-I are important activators of innate responses, as well as antigen-presenting activity, therefore playing a role in T lymphocyte activation and phenotype (23). More recently, it has become clear that human oncogenes also play a role in immune escape mechanisms (24). Stabilization of β-catenin, in the Wnt pathway, for example, reduces the expression of CCL4, a chemokine that attracts DCs, impairing tumor antigen presentation (25).

Oncogenes also drive the reprogramming of tumor cell metabolism, the so-called Warburg effect. Tumor cells display different metabolic strategies to maintain energy production and catabolism at a rate to allow continuous cell proliferation. Some cells use glycolysis almost exclusively, while others also required amino acids and fatty acids as well, and keep the Krebs cycle and oxidative phosphorylation active. In either case, tumor cells usually increase the glucose uptake and secrete lactate in higher concentrations than other cells in the body (26). Both the decrease in glucose and the increase in lactate concentration have consequences for immune responses. Activated T lymphocytes and M1 macrophages display a metabolic profile similar to tumor cells, therefore, dependent on glucose. Low glucose concentration inhibits T lymphocyte proliferation and macrophage function. Additionally, lactate is a regulatory molecule, modulating the phenotype of DCs, inducing suppressor phenotype on macrophages, and inhibiting T lymphocytes (27).

Besides tumor cell-intrinsic metabolism, other cells in the TME also display metabolic pathways that lead to tolerance. DCs, the essential population for naive T lymphocyte activation, can acquire tolerogenic phenotype due to signals from tumor cells, but also from binding, *via* CD80 or CD86, to cytotoxic T lymphocyte-associated protein 4 (CTLA-4) expressed by Treg. The signal triggered by this interaction promotes indoleamine-2,3-deoxygenase (IDO) expression in DCs. This enzyme, which physiological function is the protection of immune-privileged tissues, catabolizes the reaction that converts the essential amino acid tryptophan in kynurenine, which through the aryl hydrocarbon receptor, promotes regulatory phenotype in T lymphocytes (28). Therefore, this mechanism works as an amplification of the regulatory cycle in the TME. Furthermore, tumor cells can also overexpress IDO, as observed in oral squamous cell carcinoma from smoker patients (29). In general, IDO expression depends on IFN-*γ* stimulation, which in cancer, characterizes it as a negative feedback mechanism for effector immune responses.

Programmed cell death-ligand 1 (PD-L1) is also an inhibitory molecule expressed upon IFN-*γ* stimulation, both in antigen-presenting cells (APCs) and cancer cells (30). The receptor for PD-L1, programmed cell death-1 (PD-1), is upregulated upon T lymphocytes activation. Several transcription factors, such as NFAT, AP-1, FoxO1, and NFκB, mediate the *PDCD1* expression. Moreover, chromatin changes are also important to control PD-1 expression and are observed in exhausted CD8^+^ T cells (31). PD-1 contains immunoreceptor tyrosine-based inhibitory (ITIM) domains and can inhibit TCR signaling, rendering T cells inactive. PD-1 expressing stem-like CD8^+^ memory T cells can be found in lymphoid follicles in the tumor (32). These cells, when activated, differentiate in exhausted cells. There are two PD-1 ligands: CD274 (PD-L1), which has a basal expression in several cell types, and programmed cell death ligand-2 (PD-L2), which expression is usually limited to DCs and macrophages. A variety of cancers display constitutive PD-L1 expression, which can be triggered by genetic and epigenetic alterations in its promoter region, cytokine stimulation, such as IFN-*γ* and IL-6, growth factors, hypoxia, among others (28). The PD-1/PD-L1 signaling, which induces T cell exhaustion, is an important effect resulting from the chronicity of antigen presentation in cancer. Whenever antigens are chronically presented, negative feedback mechanisms are activated to protect the organism. This protective response is usurped by cancer to create an immune-privileged situation, and immune evasion (33).

As mentioned before, CTLA-4 binds to the co-stimulatory molecules CD80 and CD86. It competes with the activating receptor CD28, which also binds to these molecules, but with lower affinity. CD28 signaling is essential for T lymphocyte activation since it triggers the PI3K/Akt pathway and causes stabilization of the antigen activation signal. Not only CTLA-4 competes for biding to co-stimulatory molecules, but also, through a process called trans-endocytosis, this biding removes CD80 and CD86 from the APC surface, eliminating the possibility of CD28 activation, and consequently preventing T cell activity (34).

Many of these mechanisms can happen simultaneously in the TME, in a dynamic process that varies through time. To add to this complex situation, other factors should also be considered. Some tumors are very immunogenic, and tumor antigen-specific T lymphocytes can be found in the TME, where it can also be observed evasion mechanisms and chronic antigen signaling that can, eventually, inhibit anti-tumor responses. When immune responses persist, it results in chronic inflammation leading to cancer progression. Other tumors are less immunogenic and recruit mainly myeloid cells, which display a tolerogenic phenotype, helping cancer cells meet their metabolic demands, and promoting angiogenesis. M2 macrophages, for example, display arginase activity, causing conversion of arginine to ornithine, which is a substrate to the synthesis of polyamines, necessary for catabolism and cell proliferation (35).

## Autophagy Modulates Tumor Immune Environment

### Autophagy

Autophagy is a survival cellular process in which organelles and other cytoplasmic components are directed to the lysosomes for degradation (7, 36). This mechanism is highly conserved in eukaryotic cells and its activation occurs in face of starvation, hypoxia, and/or oxidative stress conditions (8). Up to now, three classes of autophagy are known: macroautophagy, microautophagy, and chaperone-mediated autophagy (CMA). In macroautophagy, an isolation membrane enclosures a portion of the cytoplasm, molecules, and organelles, forming a double-membrane vesicle associated with light chain protein-3 (LC3), called an autophagosome. LC3 is processed and cleaved, generating LC3-I, which receives carboxyl glycine radical and turns into LC3-II. LC3-II acts as a receptor in autophagosome membrane binding to p62, through the LC3-interacting region. P62 is a multidomain protein, involved in the cell death and survival process, which delivers ubiquitin radicals to LC3-II. This induces autophagosome-lysosome fusion to form an autolysosome, autophagy is then activated and p62 is degraded (37, 38). In microautophagy, an invagination of the lysosome membrane engulfs cytoplasmic compounds, in a similar process to endosome formation, producing a multivesicular body (39). Conversely, CMA is a type of autophagy used to degrade specific soluble proteins. A cytosolic substrate is recognized by the chaperone protein heat shock cognate 70 (Hsc 70), which binds to lysosomal-associated membrane protein-(LAMP) 2A in the lysosome membrane to transport this substrate into the lysosome lumen (40).

Macroautophagy is the main type of autophagy, therefore it will be referred to just as autophagy. In response to hypoxia and oxidative stress, hypoxia-inducible factors (HIF) 1 and 2 bind to hypoxia response elements (HREs), leading to the transcription of several genes that are involved in angiogenesis, metastasis, cell survival, immune escape, and autophagy pathways. Activation of HIF-1 subunit-1 leads to an increase in BCL2 interacting protein 3 (BNIP3) and BCL2 interacting protein 3 like (BNIP3L) expression levels. These factors are responsible for breaking the connection between Beclin1 (BECN1) and B-cell lymphoma 2 (Bcl-2), an inhibitory complex that prevents autophagy (41). Another way to induce autophagy in a hostile cellular environment is through the activation of autophagy-related genes (ATG) (42) and 5’-adenosine monophosphate-activated protein kinase (AMPK) (43). AMPK is a nutrient availability sensor and can regulate oxidative and glycolytic metabolism. It can also activate the autophagic recycling of cellular components to balance cellular energy supply. In autophagy activation, ATG and AMPK, independently of BNIP3 and BNIP3L, downregulate the mammalian target of rapamycin (mTOR), which drives autophagosome formation (43).

Despite being a natural process to maintain cellular homeostasis, autophagy activity has been described to contribute to the progression of many human diseases, such as some neurodegenerative disorders, infectious diseases, and cancer. In 1999, it was described that mono-allelic deletions and decrease in expression of *Beclin1*, on MCF7 human breast carcinoma cells, contributed to tumorigenesis in nude mice, indicating that autophagy could inhibit tumor growth (9). It is reasonable to assume that autophagy could stop the transformation process by eliminating oncogenic, aggregated, or erroneously folded proteins (44). Nevertheless, tumor cells and components of TME can induce autophagy to survive hostile conditions and suppress immune responses, helping tumor growth and proliferation (reviewed in 45). Thus, its role in cancer development remains unclear, and the aspects of how autophagy can modulate immune components of TME will be reviewed in the next topics.

### Autophagy and Antitumor Immune Response

#### The Dual Role of Autophagy in Antigen Presentation

**Figure 1:**
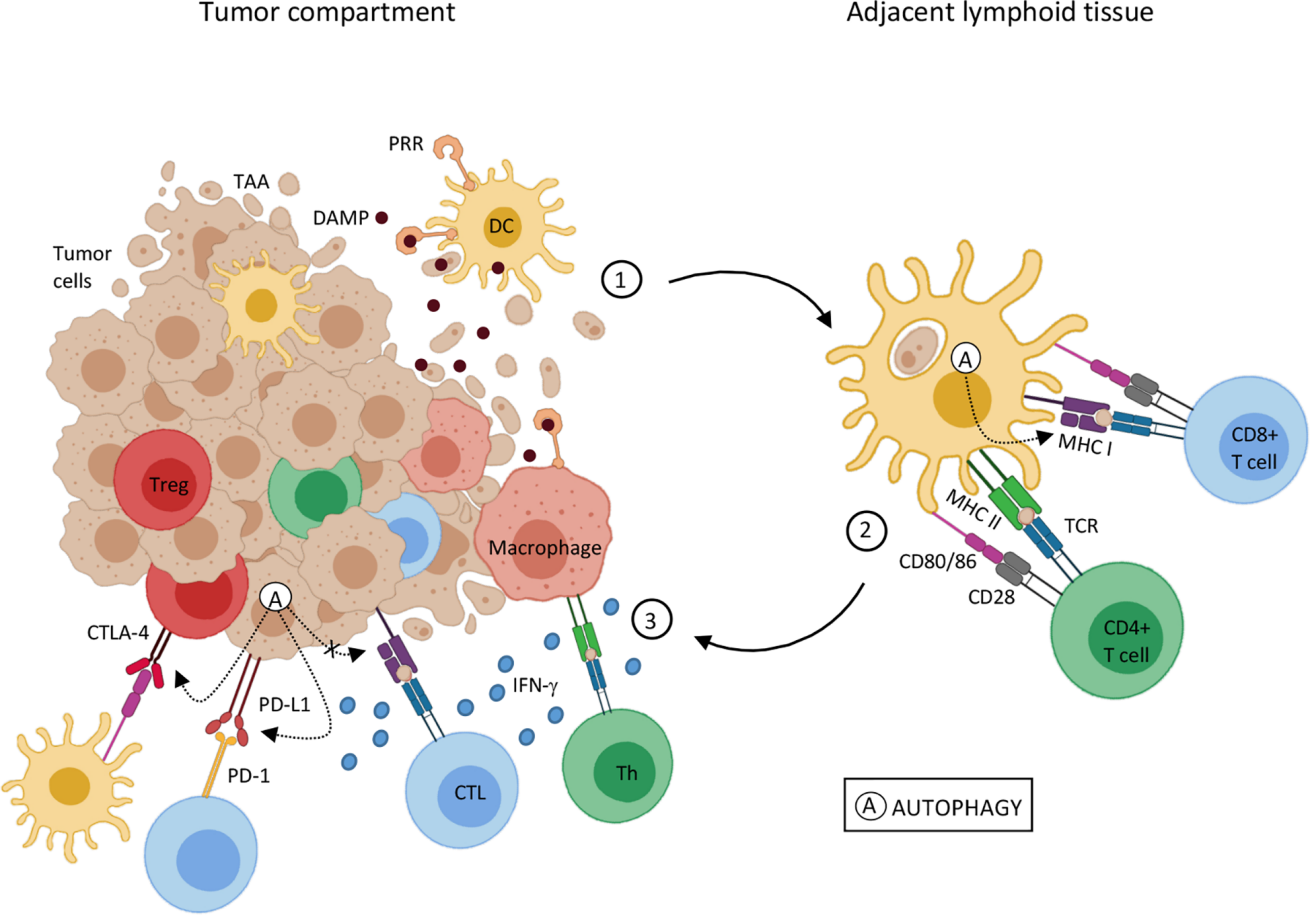
Autophagy influence on tumor-associated antigen presentation 1. Genetic alterations that cause cell transformation can also generate tumor-associated antigens (TAA) for immune recognition. Tumor cell death is an antigen source for antigen-presenting cells, such as dendritic cells (DC) and macrophages. DC activation by DAMPs and antigen processing leads to an upregulation of costimulatory molecules and MHC on DC surface, the cardinal signals for T lymphocyte activation, and migration to adjacent lymphoid tissue. 2. Mature DCs present TAA through MHC-I and MHC-II to naïve CD8+ T cells and CD4+ T cells, respectively. Antigen recognition results in T lymphocyte differentiation in effector cells (CTL e Th) and migration to the tumor site. 3. In the tumor microenvironment, upon TAA recognition through TCR interaction with MHC-I and MHC II, CTL and Th lymphocytes, respectively, trigger cytotoxic mechanisms, as interferon-gamma (IFN-*γ*) mediated ones. Despite that, inhibitory molecules, such as PD-1 and CTLA-4 in T cells, and PD-L1 in TME can interfere in T cell activation and function. IFN-*γ* stimulation can result in PD-L1 expression in both APCs and tumor cells, inhibiting T cell function. CTLA-4 expressed by regulatory T lymphocytes (Treg), through binding to co-stimulatory molecules, CD80/86, induces tolerogenic phenotype on DCs, amplifying the regulatory mechanisms in the TME. Autophagy (A) can either help or disturb the antigen presentation and T cell activation pathway. Autophagic activity on DCs seems to increase MHC-I expression, thus enhancing antigen presentation. On the other hand, autophagy activation on tumor cells may promote a reduction in MHC-I and an increase in PD-1 and CTLA-4 expression, leading to tumor progression. Sources (11, 28, 34, 47, 49, 51–56).

Studies have shown the influence of autophagy in antigen presentation (46–49), as well as in the anti-tumor adaptive immune response activation (50). As shown in **Figure 1**, adaptive anti-cancer immune responses are triggered by endogenous tumor-associated antigens (TAA) presented to T lymphocytes *via* the major histocompatibility complex (MHC) context by DCs (reviewed in (51, 53). Normally, MHC-I presents intracellular antigens, such as the ones derived from self-proteins and viral proteins, to CD8^+^ T lymphocytes while extracellular antigens are generally presented to CD4^+^ T lymphocytes by MHC-II (reviewed in 54). Effective tumor antigen presentation and the consequent effector T lymphocyte responses and access to the TME are essential for clinical responses to immunotherapy (55) and are associated with positive clinical outcomes (56). Reduction in MHC expression and expression of non-classical molecules is frequently observed in different types of cancers, leading to compromised antigen presentation and/or immune evasion (reviewed in 52), which can influence tumor progression and resistance to immunotherapy (57).

##### Effective Antitumor Immune Responses

Effective antitumor responses are dependent on potent antigen presentation and leukocyte infiltrates enriched with effector CD8^+^ T cells (49). Autophagy activation in DCs may improve antigen presentation and stimulate cytotoxic responses mediated by CD8^+^ T lymphocytes (47, 49). For example, nano-activators conjugated to antigens were used to stimulate DCs, triggering anti-tumor T cell responses in mice. It has been shown that nano-activators treated DCs, through autophagy-dependent mechanisms, could increase antigen presentation and cross-presentation to T lymphocytes, increasing effector CD8^+^ T tumor infiltrating lymphocytes (TILs) (49). Additionally, experimental data has shown that semi-synthetic vitamin E derivative alpha-tocopheryloxyacetic acid (α-TEA) could modulate autophagy in tumor cells from both Lewis lung carcinoma (LLC) and murine mammary tumor, improving antigen cross-presentation by DCs and triggering tumor antigen-specific CD8^+^ T lymphocytes responses. Treatment with α-TEA resulted in LC3-II increase, both *in vitro* and *in vivo*, indicating autophagic activity. These authors also found that α-TEA-generated autophagosome-enriched fraction (α-TAGS) was a competent tumor antigen carrier, which stimulated antigen cross-presentation mediated by DCs to CD8^+^ T cells and stimulated CD8^+^ T cell proliferation in an autophagy-dependent fashion. Overall, these findings demonstrated a new mechanism of immune activation by α-TEA, which stimulated tumor cell autophagy and antigen cross-presentation to CD8^+^ T cells (47).

Autophagy can also create new epitopes arising from stress-induced post-translational modifications, which could increase immune recognition (46). It has been shown that citrullination – the conversion of arginine residues to citrulline - can take place in cells during autophagy induced by stress and, in inflammatory conditions, it can result in MHC-II presentation of citrullinated epitopes to CD4^+^ T cells (46). Although autophagy modulation has not been directly investigated, the combination of citrullinated peptide based vaccine with TLR ligand adjuvant promoted a Th1 anti-tumor response in melanoma and ovarian cancer mouse models. CD4^+^ T TILs were associated with tumor regression. Interestingly, they also observed a Th1 response to the citrullinated peptides in ovarian cancer patients (58). Collectively, these findings indicated that autophagy is associated with efficient antigen presentation in different types of cancer. As it increases antigen availability and enhances T cell activation, it favors cytotoxic responses and clearly can act to inhibit cancer progression.

##### Immunosuppression: Autophagy Disrupts Antigen Presentation

Autophagy may also play a negative role in antigen presentation, facilitating tumor evasion from CD8^+^ T cells in both pancreatic ductal adenocarcinoma (PDAC) and melanoma (11, 59). PDAC displays low levels of MHC-I surface molecules. In these tumor cells, knockdown of the autophagy cargo receptor gene, *NBR1*, increased MHC-I surface expression, confirming the implication of NBR1-mediated autophagy-lysosomal pathway in the process. The authors showed that NBR1 targeted MHC-I for lysosomal degradation. Mouse PDAC cells expressing an autophagy inhibitor, restored MHC-I membrane expression, improved antigen presentation, and CD8^+^ TILs, leading to a reduction in tumor growth. These findings indicated that high levels of MHC-I at PDAC cell surface after autophagy inhibition were required to increase CD8^+^ T cell infiltration and to kill the tumor cells (11).

The activation of autophagy pathways has also been described in macrophages and DCs infiltrating B16F10 mouse melanoma. These tumors normally express T cell immunoglobulin and mucin domain protein-4 (TIM-4). Autophagy initiates when TIM-4 binds to AMPK-α1. This activation promotes the degradation of TAA through the lysosomal pathway, which led to a decrease in antigen presentation and, consequently, in specific anti-tumor CD8^+^ T cells. TIM-4 blockade with a monoclonal specific antibody resulted in autophagy inhibition and improvement in antigen cross-presentation and IFN-*γ* production (59). Moreover, chloroquine (CQ), a known autophagy inhibitor, combined with low concentrations of 5-fluorouracil (5-FU), increased DCs maturation and activation in HCT-116 colorectal cancer cells, enhancing CD8^+^ T lymphocyte stimulation (60). Together, these results suggested that autophagy can impair antigen presentation by interfering with different key steps in this process.

#### TME Induces Autophagy

**Figure 2:**
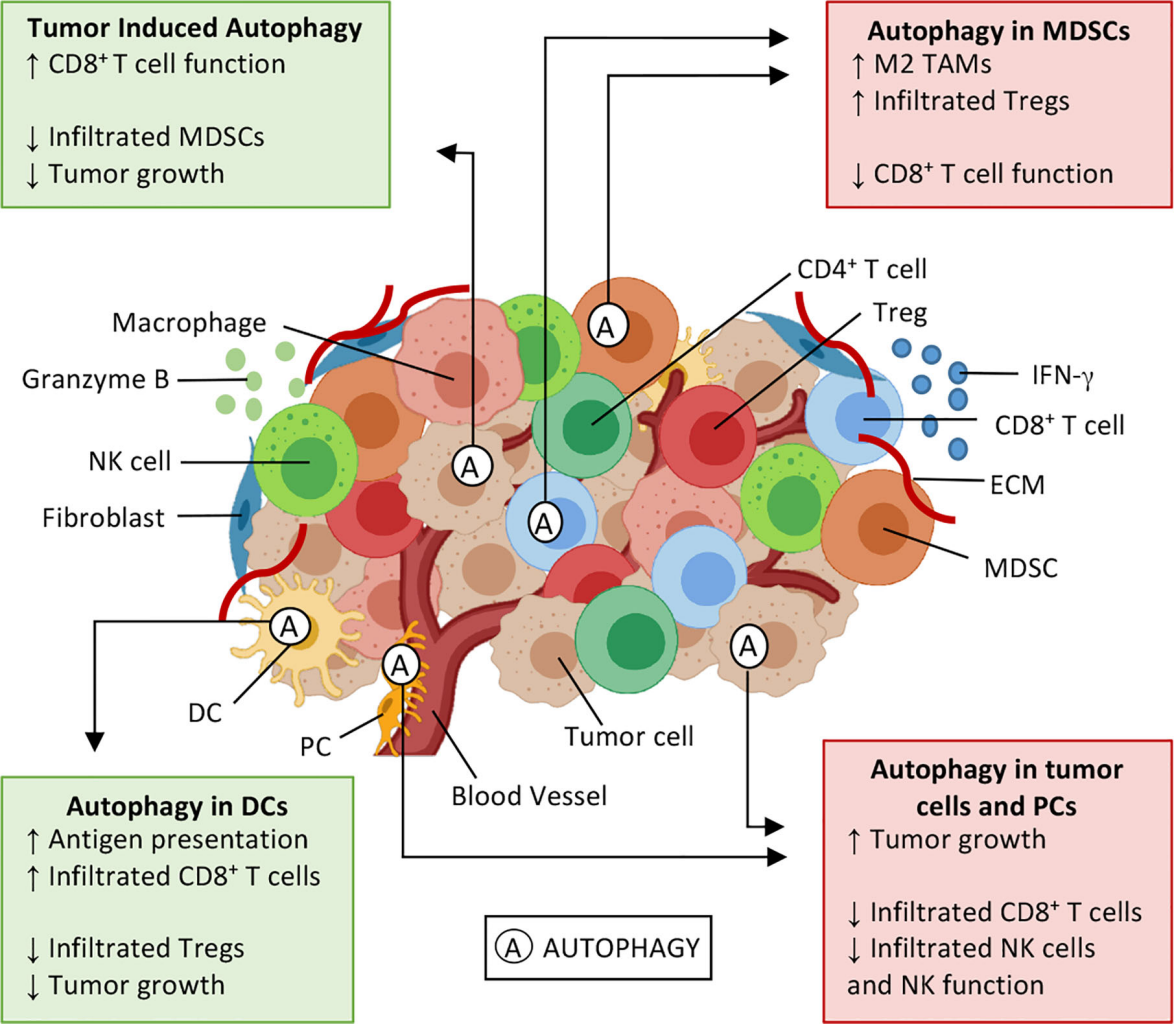
Effects of autophagy on the tumor microenvironment. Tumor microenvironment (TME) is composed by cytokines (e.g. IFN-*γ*), extracellular matrix (ECM), and several cell types: tumor cells, fibroblasts, and immune cells, such as natural killer (NK) cells, CD8^+^ T and T helper (Th) lymphocytes, T regulatory (Treg) cells, myeloid-derived suppressor cells (MDSC), dendritic cells (DCs), macrophages, and pericytes (PC). Autophagy (A) is a cellular survival mechanism, which is activated in stressful conditions, can be activated in TME. Autophagy can either enhance antitumor immune responses (green boxes) or induce an immunosuppressive environment (red boxes), thus playing a dual role in cancer development and progression. Autophagy activation in DCs enhances antigen presentation and results in an enrichment of CD8^+^ T tumor-infiltrating lymphocytes, also decreasing infiltrated Treg and tumor cell proliferation. Similar outcomes can be seen when IFN-*γ* induces autophagy on tumor cells, and elevated extracellular potassium induces autophagy on CD8^+^ T cells. In these situations, it is possible to observe a decrease in infiltrated MDSCs and T lymphocytes expressing PD-1, thus inhibiting tumor growth. Contrarily, the autophagy activation in myeloid cells and tumor cells has the opposite effect, favoring an immunosuppressive profile of TME, leading to tumor cell proliferation, through M2 macrophage polarization, enhancing Treg infiltration and inhibitory molecules (PD-1 and CTLA-4) expression. Sources (5, 6, 11, 47, 49, 61–67).

Not only autophagy modulates TME components, but the opposite is also true. As shown in **Figure 2**, cytokines and metabolic conditions may also promote or inhibit autophagy and influence the tumor immune response. IFN-*γ* can induce autophagy in gastric cells, thus inhibiting carcinogenesis (61). Gastric cancer is usually associated with chronic inflammation. Transgenic mice that overexpress IFN-*γ* in the gastric mucosa (H^+^/K^+^-ATPase-IFN-*γ* and H^+^/K^+^-ATPase-IL-1β; IFN-*γ*) displayed protection against gastric dysplasia in comparison to controls. The authors observed that gastric cells displayed lower proliferation rates and T cell apoptosis dependent on IFN-*γ* expression. Furthermore, transgenes resulted in increased levels of LC3-II and Beclin-1 mRNA and protein, in the stomach, indicating autophagy activation. Additionally, transgenic animals showed higher apoptotic T cells, concurrently with inhibition of IL-6, IL-1β; and TNF-α production, and presented less chronic inflammation (61).

Besides the cellular components and soluble mediators, TME also display metabolic disorders such as aerobic glycolysis (68), oxygen deprivation, and higher levels of extracellular potassium (62), which are stressful conditions that can also influence cellular autophagy. Activation of autophagy in T lymphocytes exposed to elevated potassium concentrations reduced acetylation of the promoter and enhancer regions of T cell effector- and exhaustion-markers *Ifng* and *Pdcd1.* Potassium exposed T lymphocytes adoptively transferred to B16 melanoma bearing mice resulted in T cells persistence in the TME, driving tumor regression, and improving animal survival (62). Furthermore, metabolic conditions of TME, such as elevated extracellular potassium levels, were responsible for autophagy activation on T-cells and boosted antitumor responses (62).

Despite that, tumor metabolism can play tricks on antitumor immune responses, such as the ones with elevated glycolytic metabolism, that seems to inhibit autophagy pathways and fuel MDSCs development, leading to an immunosuppressive environment (68). MDSCs and Treg cells suppress T lymphocyte effector functions in the TME and are enriched in tumors with elevated glycolytic metabolism, such as triple-negative breast cancer (TNBC). These tumors secrete high levels of G-CSF, which stimulates MDSCs, and lower expression levels of LC3 mRNA. Inhibition of the glycolytic enzyme lactate dehydrogenase A (LDHA) in 4T1 and Py8119 TNBC cells using target-directed short-hairpin(sh), restored tumor autophagy, and reduced MDSC infiltration in tumors growing in BALB/C and C57/BL6 mice, respectively. The mechanism was dependent on AMPK-ULK1 signaling, which was impaired by glycolysis. At the same time, glycolysis and AMPK-ULK1 inhibition increased G-CSF expression. Consequently decreasing IFN-*γ* ^+^ and TNF-α^+^ effector CD8^+^ T TILs and in tumor-draining lymph nodes, resulting in an immunosuppressive environment. Consequently, autophagy boosted antitumor response mediated by effector CD8^+^ T cells (68). These results described above indicate that autophagy may be an important mechanism in tumor immune responses. Sometimes, directly improving the cytotoxic activity of T cells, and others, modulating the immunosuppressive components of TME, such as infiltrated MDSCs and T-cell exhaustion-markers, leading to tumor elimination and better survival rates.

### Autophagy and Immune Evasion Mechanisms

Despite the evidence that autophagy can improve antitumor immune responses, it can also inhibit both innate and adaptive responses, leading to cancer immune evasion.

#### Autophagy in Myeloid Cells Induces an Immunosuppressive TME

It is known that MDSCs and M2 macrophages are components of the immunosuppressive compartment of TME. The autophagic activity in MDSCs has been described by different research groups (63, 64) and has been associated with antigen presentation and cytotoxic T cell function impairment, as well as to M2 macrophage polarization and Treg recruitment (**Figure 2**) (48, 63).

The importance of a non-canonical autophagy pathway, the LC3-associated phagocytosis (LAP), in TME was demonstrated using Cre-lox based ablation of several genes in myeloid cells of immunocompetent mice (C57BL/6). The ablation of *Becn1*, *Vps34*, *Atg5*, *Atg7*, or *Atg16l1* impacted on both conventional autophagy and LAP pathway. While myeloid cells deficient in *Fip200*, *Ulk1*, or *Atg14* lacked only the canonical autophagy pathway, and the absence of *Rubicon* or *Nox2* affected only the LAP pathway (63). In the LLC mouse model, the absence of LAP (*Rubcn* ^-/-^) in myeloid cells increased the co-stimulatory molecule CD86 and reduced CD206, a mannose receptor associated with M2-like phenotype. In this model, the reduction on M2-like TAMs improved CD8^+^ and CD4^+^ T cell IFN-*γ* production, increased IFN-I, and IL-1β, although no quantitative alteration in the frequency of TILs was observed (63), suggesting modulation of T cell activity rather than proliferation or recruitment.

Similarly, an increase in autophagy activity was observed in MDSCs from melanoma patients and melanoma experimental model. Functional autophagy was measured by the expression of LC3, LAMP-1, and SQSTM1/p62, and colocalization analysis with p62 and LC3 in MDSCs isolated from melanoma patients (stages III and IV) and clinically healthy controls’ peripheral blood. MDSCs from melanoma patients, and also from mice with melanoma, showed higher levels of functional autophagy. In LysM^cre^ *Atg5* ^fl/fl^ mice, in which myeloid cells lacked *Atg5* expression, there were less Treg TILs, tumor cell proliferation rate reduction, and a significant increase in both MHC-II expression and IFN-*γ* production (64). Again, an indication that autophagy activity in TME myeloid cells plays a role in these cells’ immunosuppressive functions.

#### Autophagy Disrupts the Cytotoxic Activity of TME

TME is a hostile environment where autophagy can lead to degradation of cytotoxic molecules (granzyme B and IFN-*γ*), expression of T cell exhaustion markers, and quantitative changes in TILs.

NK cells release granules containing perforins and granzymes as part of their effector mechanism. In MCF7 breast cancer cells, granzyme B suffered lysosome degradation, after hypoxia-induced autophagy, which inhibited NK cell-mediated tumor cell lysis (65). Likewise, *BECN1* inhibition increased functional NK cell tumor infiltration in a melanoma mouse model. The higher frequency in infiltrating NK cells was correlated with an increase in CCL5 secretion, which is an important chemokine for NK cell proliferation and activation. The enhancement of NK cell function, after inhibition of tumor-autophagy, caused tumor regression and predicted improved patient survival (66), suggesting that tumor cell-autophagy can be a resistance mechanism to NK cell activity.

The impact of autophagy in cytotoxic immune responses also influences CD8^+^ and CD4^+^ T cells, although there is no consensus about this topic in the literature. *Atg5* deficient mice displayed a reduction of CD8^+^ TILs in mammary and colorectal cancer models. Despite that, among the CD8^+^ T lymphocytes recruited to the tumors, around 80% exhibited memory phenotype and were positive for IFN-*γ* and TNF-α, leading to tumor rejection (69). Moreover, in the non-small cell lung cancer (NSCLC) mouse model, *Atg5* tissue-specific depletion promoted tumor initiation through a mechanism dependent on Treg recruitment to the TME (48). These findings indicated that autophagy may have a positive effect on cytotoxic immune responses, through the reduction in Treg frequency in the TME and increase in IFN-*γ* and TNF-α release.

On the other hand, the interaction with human glioblastoma (GB) cells induced *Lamp2a* mRNA and protein expression in mouse brain pericytes (PC). Engraftment of PC from *Lamp2a* knockout mice (PC-KO) into GB grown in mice led to central-memory (CD44^+^, CD62^+^) CD4^+^ and CD8^+^ T TILs and better CD4^+^/CD8^+^ ratio of 2:1 than in tumors engrafted with wild type (WT) PC. Moreover, only 2% of TILs expressed PD-1 and CTLA-4 and there was a lower GB cell proliferation rate than in GB/PC-WT control mice. Compared to GB/PC-KO, GB/PC-WT mice displayed increased IL-2 levels, tumor proliferation, FoxP3^+^PD-1^+^CTLA-4^+^ TILs, higher *Tgfβ*, and *Il10* mRNA expression levels, and a CD4^+^/CD8^+^ ratio of 4:1. Collectively, these results demonstrated that CMA activity in PC favored an immunosuppressive environment in response to GB cells (64).

Inhibitory molecules expression is associated with CD8^+^ T cell exhaustion phenotype, and also resistance to targeted therapy and autophagy inhibitors. PD-L1^+^ A375 melanoma cells showed resistance to BRAF inhibitor Vemurafenib and CQ, but not to nitrobenzoxadiazole (NBD). Both CQ and NBD inhibit autophagy, however, NBD acts at multiple levels, targeting not only the late stages of autophagy but also different apoptotic pathways (70). These findings indicate that autophagy may act as an immunosuppressive mechanism, affecting cytotoxic cells—NK and T lymphocytes—functionally and quantitatively. Additionally, autophagy modulates immune checkpoints expression, leading to T cell exhaustion phenotype and resistance to treatment.

## Autophagy and Cancer Therapy

Autophagy can be a preventive mechanism to malignant transformation in healthy cells, but in transformed cells this mechanism can contribute to cancer progression. Autophagy’s main roles in tumorigenesis and cancer therapy were reviewed elsewhere (71). In this section, we will focus on the interplay between autophagy and immune responses in cancer therapies in different experimental models.

### Autophagy and Conventional Therapies

Traditionally, cancer therapy is based on direct toxicity to tumor cells. But the importance of immune responses to the efficacy of traditional therapies can be observed in mouse models, in which T lymphocytes are necessary for tumor growth reduction by chemotherapeutic agents (72). Recently, it has become clear that several chemotherapeutic agents, such as doxorubicin and mitoxantrone (MTX), and even radiotherapy, may induce tumor cell immunogenic cell death (ICD) (72–74). ICD is characterized by the release of damage-associated molecular patterns (DAMPs) and consequent elicitation of immune responses. The pre-apoptotic surface exposure of calreticulin (CRT) is considered the “eat me” (immunogenicity) signal for DCs, influencing antigen presentation to T lymphocytes (73). Likewise, adenosine triphosphate (ATP) secreted or leaked to the extracellular milieu is considered a “find me” signal. The chromatin-binding protein high mobility group B1 (HMGB1) is another DAMP exposed after chemotherapy by post-apoptotic release. It induces TLR4-MyD88 signaling on DCs facilitating antigen processing and presentation (74, 75).

The ATP lysosomal secretion depends on autophagy, as autophagy-deficient CT26 colon carcinoma mouse cells (due to the lack or decrease in *Atg5* or *Atg7* expression) released lower amounts of ATP in response to MTX chemotherapy. The lower ATP release resulted in DCs recruitment impairment and consequent lack of T cell priming to elicit an anti-tumor immune response (76). Extracellular ATP binds to surface purinergic receptors (such as P2YR2 receptors) on immature DC precursors (77), promoting DC maturation and recruitment to the tumor site in lung cancer mouse model. In line with that, autophagy induction (by fasting or caloric restriction) resulted in ATP release and improved chemotherapy “efficacy” by decreasing TME infiltration by Treg (78).

CD39, an ectonucleotidase, also influences the extracellular ATP concentration by converting extracellular ATP into adenosine. CD39 overexpression was observed in *Atg5* deficient tumors, leading to the attraction of Treg expressing adenosinergic receptors to the TME (48). Indeed, an enhanced number of initial tumor foci and increased Treg infiltration were observed in autophagy-deficient tumors in the KRasG12D-driven lung cancer mouse model (48). In contrast, similar T cell infiltration and function in autophagy-deficient (due to inhibition of autophagy essential genes *Atg7* and *Atg12*) or competent tumors were observed in the B16 melanoma mouse model, even after Doxorubicin treatment (10). These data suggest that autophagy-dependent immune modulation may be specific to the clinical context and time (10). However, the complexity of autophagy role in carcinogenesis can be seen in the KRasG12D-driven lung cancer mouse model, because mice with autophagy-deficient-tumors presented a prolonged survival and reduced malignant progression of adenomas to adenocarcinomas, in a TP53 dependent manner. Although the autophagy modulation needs to be investigated in each case, these data indicated that tumor-specific loss of *Atg5* favored Treg TILs. Thus, autophagy-deficient tumors (or with CD39 overexpression) treated with ICD inducer chemotherapy agents did not recruit effector cells and it possibly contributed to chemotherapy (CT) resistance (48).

Indeed, there is evidence that autophagy may play a role in CT resistance in pancreatic cancer patients. A clinical trial to investigate if autophagy inhibition by HCQ (Hydroxychloroquine sulfate) improved overall survival of metastatic pancreatic cancer patients treated with gemcitabine hydrochloride and nab-paclitaxel was performed (79). Autophagy inhibition did not improve 1-year patients’ overall survival, but there was an improvement in the overall response rate in HCQ treated patients, indicating its role in the locally advanced setting, supporting the need for more research and biomarkers to drive this therapeutic option. Likewise, the treatment of bladder cancer cells (J82 and T24) with enzalutamide, an anti-androgen receptor drug, resulted in cytoplasmatic autophagosomes accumulation, increased expression of autophagy-related genes (*AMPK*, *ATG5, LC3B*, *ULK1*, and *LC3-II*) and had no effect on apoptosis and proliferation rates. However, the treatment with a combination of enzalutamide and autophagy inhibitors (CQ, 3-methyladenine, and bafilomycin A1) impaired tumor growth, indicating that the combined treatment may be a potential strategy to avoid enzalutamide-resistant bladder cancer (80). A similar result was exhibited in docetaxel resistant prostate cancer cell lines (PC3-DR and VCaP-DR): these cells present enhanced autophagy activity through the overexpression of Forkhead box protein M1 (*FOXM1*). Thus, the knockout of *ATG7*, *Beclin-1*, or treatment with CQ restored the antitumor effect of docetaxel, demonstrating that either autophagy or *FOXM1* may be potential targets for combined therapies with docetaxel to treat metastatic prostate cancer patients (81). On the other hand, new therapies may be used to activate autophagy, improving the efficacy of conventional treatment, such as CT. Oxaliplatin-induced ICD was not sufficient to completely eliminate both breast and colorectal tumors (82, 83). Oxaliplatin treatment together with a nanoparticle in CT26 tumor-bearing mice led to autophagy activation on tumor cells, improving antigen presentation, and consequently tumor cell death (83).

The role of autophagy in other cells beyond the tumor ones was investigated to elucidate the hypothesis that autophagy competence in the immune system would contribute to the reduction of tumor growth by ICD-inducing CT. However, the growth of autophagy competent tumors was the same in wild type and partially autophagy-deficient (*Becn1*± or *Atg4b* ^-/-^) mice, although the MTX toxicity was higher on *Atg4b* ^-/-^ mice (84). These results pointed to an autophagy role in tumor cells influencing anticancer immune responses induced by CT. Nonetheless, a similar T cell profile was observed in the B16 melanoma mouse model, both in tumor-specific autophagy inhibition treated with Doxorubicin (10) and in systemic inhibition of autophagy with CQ and quinacrine, suggesting that host autophagy competence did not influence the efficacy of ICD-inducing CT in this model (10).

Response to radiotherapy may also be influenced by autophagy. Radiation therapy induced MHC-I expression in NSCLC cell lines A549 and H1975 in parallel with an increase in the LC3-II/LC3-I ratio, while p62 detection decreased (85). Treatment of both cell lines with autophagy inhibitor CQ after radiation resulted in decreased MHC-I expression, indicating that radiation-induced MHC-I expression and CD8^+^ T cell infiltration were dependent on autophagy (85). It was also demonstrated that imiquimod (TLR7 agonist) activated oxidative stress, inducing autophagy, and sensibilization of melanoma cells to *γ*-ionizing radiation (86). Mouse treatment with 3-MA, an autophagy inhibitor, after radiation restored the B16F10 or B16F1 tumor growth. The combined imiquimod and radiation therapy increased IFN-*γ* and TNF-α secreting CD8^+^ T lymphocytes and decreased Treg and MDSCs in the TME (86), indicating the influence of autophagy in the regulation of therapy-induced immune responses.

### Autophagy Improves New Therapeutic Strategies, by Modulating TME Components

Recently, immunotherapy has emerged as a therapeutic option able to elicit high rates of durable anti-tumor responses in cancer patients, including the ones with previously refractory responses to CRT (87). Immunotherapy aims to activate and recruit immune cells to the TME to target transformed cells. The best results have been achieved in patients with immunogenic tumors, which express high levels of neoantigens, such as metastatic melanoma. Even so, a proportion of patients with no clinical benefit after immune checkpoint blockade therapy has been reported in several studies, and autophagy may play a role in this resistance to therapy. A set of melanoma-associated antigen (MAGE) cancer-germline antigens was identified as a predictor of CTLA-4 blockade resistance in melanoma patients (88). Interestingly, autophagy markers, including LC3B, were enriched in MAGE negative tumors, suggesting that autophagy suppression (in MAGE positive tumors) may contribute to resistance to CTLA-4 inhibitor therapy (88). Another autophagy mechanism that influences the TME was observed in head and neck squamous cell carcinoma (HNSCC). Despite the high immune cell infiltration in HNSCC, response rates to immune checkpoint blockade therapy are low. It was identified that the SOX2 oncoprotein elicited an autophagy-dependent degradation of STING, which mediates IFN-I activation, important for Th1 chemokines production and M1-like macrophage polarization (89). In mice with HNSCC, the immunosuppressive TME could be reversed by vaccination with nanosatellite SatVax, which enhanced the potency of STING agonist and delivered high-density tumor antigens, improving tumor-specific T cell infiltration. Associated with anti-PD-L1 therapy, it could prevent the CD8^+^ T cell exhaustion (the therapy expanded CTL effectors and reduces the CD8^+^ T cells exhausted) (89).

Along with that, autophagy-deficient 4T1 cells, through *Atg5* or *Beclin1* depletion with specific single guide(sg)RNA, generated larger tumors with reduced CD4^+^ and CD8^+^ TILs and IFN*γ* ^+^ T cells in Balb/c mice, when compared to autophagy competent 4T1 cells. Moreover, the antitumor effect of the anti-PD1 antibody was limited in autophagy-deficient tumors, while a significant reduction in tumor volume and increased cytotoxic activity of TILs was observed in the control group (90). Similarly, a combination of anti-PD1 immunotherapy with anti-angiogenic endostatin, Endostar, promoted the activation of autophagy pathway PI3K/AKT/mTOR in LLC-bearing mice. Combined therapy suppressed tumor growth and modulated TME, decreasing IL-17 and TGFβ1 while reducing infiltrated MDSCs and reversing CD8^+^ T cell suppression (91). These results indicated that the complex relationship between autophagy and TME components may be important not only to the modulation of immune responses but also to define treatment efficacy and resistance.

In addition to immunotherapy, a new therapeutic approach using RP-182, a synthetic peptide analog to naturally occurring antimicrobial peptides, triggered a conformational switch in the mannose receptor CD206, M2 macrophage marker. It resulted in endocytosis, autophagy, and apoptosis of these cells and also a shift toward an M1 phenotype in the remaining cells. This treatment enriched M1-like macrophages in TME and increased the antitumor immune response in the pancreatic cancer animal model, as well as CT-26 and B16 models (92).

## Conclusions

In conclusion, we described data suggesting that autophagic activity plays a dual role in cancer development and progression, modulating TME in many different ways, that can either help or inhibit tumor development, as shown in **Figure 2**. DC-autophagy and TME-induced autophagy are usually associated with better antitumor responses and improvement of antigen presentation and cytotoxic activity, inhibiting regulatory T lymphocytes and MDSCs. However, myeloid cells and tumor cells autophagy seem to have the opposite effect. It improves immunosuppressive TME, through the recruitment of MDSCs and M2-like macrophage polarization, leading to tumor progression and worst prognosis. The influence of autophagy also reaches cancer treatment. The activation of autophagy pathways modulates TME by inducing macrophage polarization (M1-like phenotype), reducing CD8^+^ T cell exhaustion and Tregs infiltration. Therefore, targeting autophagy could improve ICD-induced by conventional and non-conventional therapies.

## Author Contributions

This review was drafted by AS, LG, APL, and PA-S, then critically revised by PA-S. Figures were elaborated by AS and PA-S. All authors contributed to the article and approved the submitted version.

## Funding

We thank the Conselho Nacional de Desenvolvimento Científico e Tecnológico (CNPq) for the scholarship provided to LG (141955/2020-1) and fellowship to AL (307841/2018-9), and the Coordenação de Aperfeiçoamento de Pessoal de Nível Superior (CAPES) for the scholarship provided to AS (88882.382114/2019-01). This work was partially funded by LIGH-FUNPAR Alliance.

## Conflict of Interest

The authors declare that the research was conducted in the absence of any commercial or financial relationships that could be construed as a potential conflict of interest.

## Abbreviations

IFN-*γ*; interferon gamma; TME, tumor microenvironment; ECM, extracellular matrix; NK, natural killer; TAMs, tumor-associated macrophages; MDSC, myeloid-derived suppressor cell; ROS, reactive oxygen species; AIDS, Acquired Immune Deficiency Syndrome; HAART, highly active antiretroviral therapy; IL, interleukin; Th, T helper; KIR, killer immunoglobulin-like inhibitory receptor; HLA, human leucocyte antigen; TCR, T cell receptor; DC, dendritic cell; Treg, regulatory T cells; IRF1, interferon regulatory factor 1; HDAC, histone deacetylase; IFN-I, type I interferon; TNF-α, tumor necrosis factor alpha; CTLA-4, cytotoxic T lymphocyte associated protein 4; IDO, indoleamine-2,3-deoxygenase; PD-L1, Programmed cell death-ligand 1; APC, antigen presenting cell; PD-1, programmed cell death-1; ITIM, immunoreceptor tyrosine-based inhibitory; PD-L2, programmed cell death ligand-2; CMA, chaperone-mediated autophagy; LC3, light chain protein-3; Hsc 70, heat shock cognate 70; LAMP, lysosomal-associated membrane protein; HIF, hypoxia-inducible factor; HREs, hypoxia response elements; BNIP3, BCL2 interacting protein 3; BNIP3L, BCL2 interacting protein 3 like; Bcl-2, B-cell lymphoma 2; ATG, autophagy-related genes; AMPK, adenosine monophosphate-activated protein kinase; mTOR, mammalian target of rapamycin; TAA, tumor-associated antigens; MHC, major histocompatibility complex; TILs, tumor infiltrate lymphocytes; α-TEA, alpha-tocopheryloxyacetic acid; LLC, Lewis lung carcinoma; TLR, toll-like receptor; PDAC, pancreatic ductal adenocarcinoma; TIM-4, T cell immunoglobulin and mucin domain protein-4; CQ, chloroquine; TNBC, triple negative breast cancer; LDHA, lactate dehydrogenase A; G-CSF, granulocyte colony- stimulating factor; LAP, LC3-associated phagocytosis; NSCLC, non-small cell lung cancer; GB, glioblastoma; PC, pericytes; NBD, nitrobenzoxadiazole; MTX, mitoxantrone; ICD, immunogenic cell death; DAMPs, damage-associated molecular patterns; CRT, calreticulin; ATP, adenosine triphosphate; CT, chemotherapy; HCQ, Hydroxychloroquine sulfate; MAGE, melanoma-associated antigen; HNSCC, head and neck squamous cell carcinoma.
